# Spatial Uncertainty Modeling of Fuzzy Information in Images for Pattern Classification

**DOI:** 10.1371/journal.pone.0105075

**Published:** 2014-08-26

**Authors:** Tuan D. Pham

**Affiliations:** Aizu Research Cluster for Medical Engineering and Informatics, Center for Advanced Information Science and Technology, The University of Aizu, Aizu-Wakamatsu, Fukushima, Japan; University of Manchester, United Kingdom

## Abstract

The modeling of the spatial distribution of image properties is important for many pattern recognition problems in science and engineering. Mathematical methods are needed to quantify the variability of this spatial distribution based on which a decision of classification can be made in an optimal sense. However, image properties are often subject to uncertainty due to both incomplete and imprecise information. This paper presents an integrated approach for estimating the spatial uncertainty of vagueness in images using the theory of geostatistics and the calculus of probability measures of fuzzy events. Such a model for the quantification of spatial uncertainty is utilized as a new image feature extraction method, based on which classifiers can be trained to perform the task of pattern recognition. Applications of the proposed algorithm to the classification of various types of image data suggest the usefulness of the proposed uncertainty modeling technique for texture feature extraction.

## Introduction

There are different types of images used in the diverse applications of image classification; to name a few, medical images, biological images, remote-sensing images, scene images, and so on. The information contents of different image types are different from each other, but they may share some common properties. To classify categorical images, the numerical description of the images, which is known as an image feature, is necessary in order to capture their distinctive characteristics that can be used for training classifiers or as labeled samples. A critical challenge in the discriminative quantification of image properties of various regions of interest is that they are usually subject to noise and vague boundaries between the objects and background, which are often found in medical, life-science, and natural data [1]–[7]. Consequently, these factors adversely affect the classification performance. To deal with the uncertainty of image information, statistical measures of sets of images are often utilized to construct probability models of images, in which they are considered as random variables. An approach for handling imprecision rather than randomness in images is to consider them as fuzzy events so that some non-probabilistic measure of uncertainty can be established.

**Figure 1:**
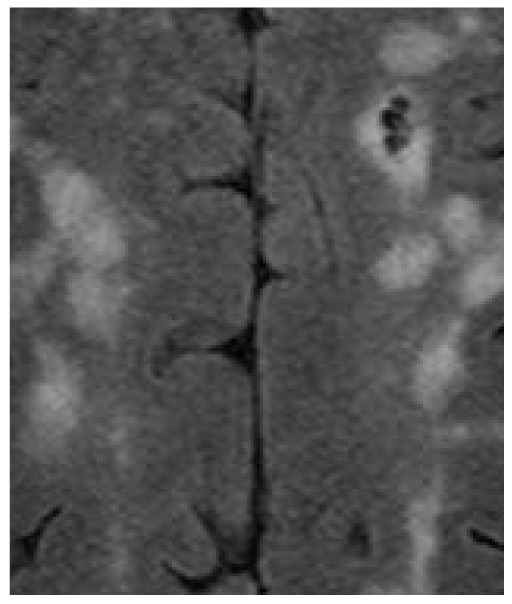
Image showing part of an MRI of the brain: bright patches are the white matter hyperintensities or lesions of the brain, and other parts are brain tissues and fluid.

**Figure 2:**
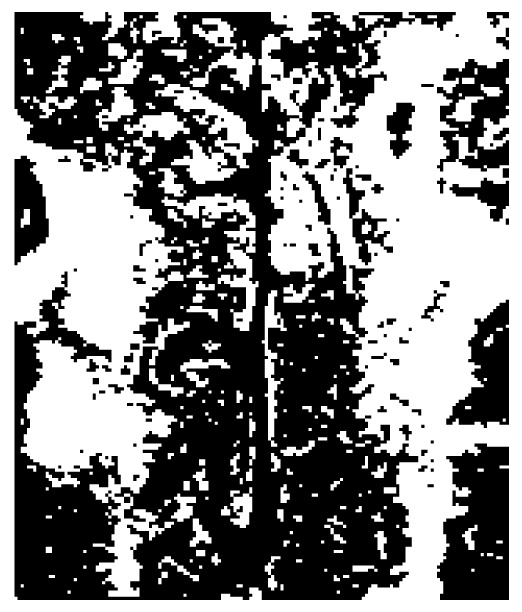
Binary segmentation of the brain MRI using gray-level threshold of 0.3.

**Figure 3:**
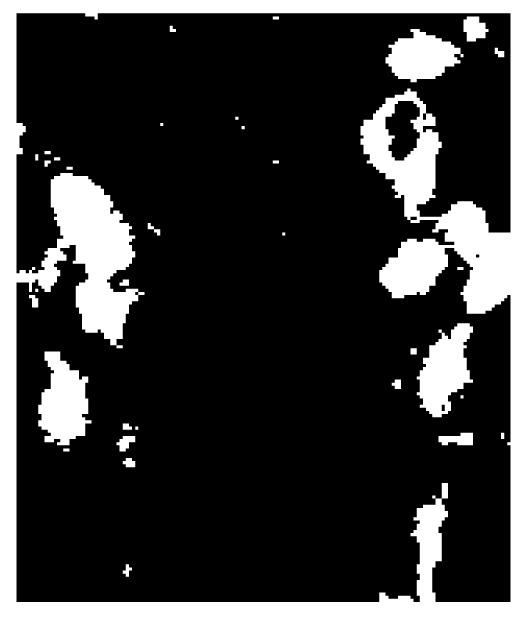
Binary segmentation of the brain MRI using gray-level threshold of 0.4.

**Figure 4:**
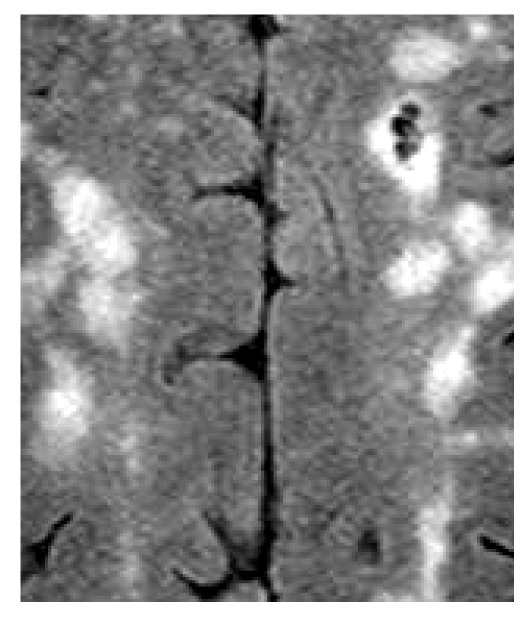
Enhancement of the brain MRI using contrast adjustment.

**Figure 5:**
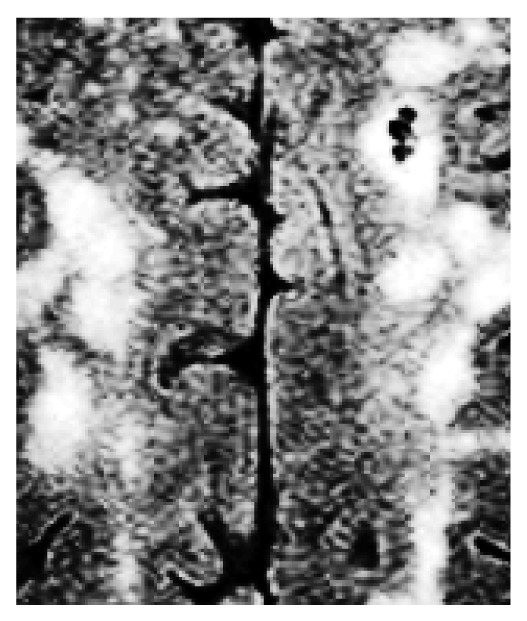
Enhancement of the brain MRI using histogram equalization.

Based on the example made to interpret the definition of the entropy of fuzzy sets [8], the above notions of uncertainty in images can be elucidated by considering examples of the outcomes of image segmentation and image enhancement. Figure 1 shows an original part of an MRI image of the brain, in which the bright areas are the white matter hyperintensities of the brain. The image intensity levels are in the range [0, 1]. Figures 2 and 3 are the two binary segmentation results of the original image (Figure 1) using the gray-level threshold of 0.1647 obtained from the Otsu's image segmentation method [9] (there are well-known public-domain software packages for MRI brain segmentation, which include prior anatomical information, such as SPM [10] and its latest version SPM8, and FSL [11]; this segmentation method used here to only provide a simple example of a segmented section of the brain on MRI for the conceptual discussion in this study), and by setting the threshold at 0.25, respectively. Figures 4–5 shows an enhancement result of the original image using an adjustment method that increases the contrast of the image by mapping the values of the original intensity image to new values such that 1% of the data is saturated at low and high intensities of the input data. Figure 5 is another enhancement result of the original image using histogram equalization that enhances the contrast of the image by transforming the values in the original image so that the histogram of the output image approximately matches the uniform distribution histogram. The pixel intensity in Figures 2 and 3 are either black (background) or white (object). In other words, the uncertainty involves in the segmentation outcomes refer to the presence or absence of object pixels, which can be modeled as a random variable taking on the values of either 1 (white object) or 0 (black background). The uncertainty associated with the outcomes shown in Figures 4 and 5 can be expressed in terms of the degree of grayness of the pixels that are subject to imprecision or subjectiveness. Therefore, such a description of imprecise pixel values can be considered as a fuzzy set. In fact, image enhancement is the process that attempts to improve an image appearance so that it looks subjectively better. There exist no standards that can guide how the image should look, but one can tell if it has been improved or not by considering, for example, the detailed contents or contrast of the image [12].

The treatment of uncertainty in images have been largely discussed using the theory of fuzzy sets [13], [14] and geostatistics [15], [16]. Fuzzy logic addresses uncertainty that is caused by imprecision or vagueness in provided information and models such uncertainty with the admission of degrees of possibility. By using the notion of fuzzy sets, an event, which is a set of outcomes of an experiment to which a probability is assigned, can be extended to define fuzzy events to significantly enhance the applications of the theory of probability in the fields in which uncertainty being due to fuzziness is inherently pervasive [17]. Although the integration of the theories of probability and fuzzy sets is natural in image analysis and pattern recognition, little effort has been spent on exploring the potential application of this idea since a work on binary image thresholding carried out by extending the probability measure of fuzzy events to calculate the fuzzy measure of similarity between two sets [18].

Furthermore, the concept of modeling uncertainty associates different sources of uncertainty with various deterministic and non-deterministic laws of physical and dynamical processes, depending on the purpose for which the models are applied. Many pattern classification problems, such as image analysis, involve spatial modeling that naturally calls for techniques covered in geostatistics. In fact, the variability in images with alternating high and low pixel values is evident in many practical domains of medicine, science and engineering. Modeling spatial continuity in images is therefore critical to providing a solution to the question of addressing uncertainty, since a spatial model of image properties will constitute to a different assessment of uncertainty compared with the assumption that everything is random [19]. Research works on geostatistics have been reported in literature, including ordinary kriging and indicator kriging, applied to image processing [16], [20] and classification of remote-sensing images [21]–[23].

The combinations of geostatsitics, fuzzy sets and fuzzy cluster analysis have been developed for image enhancement [15] and image segmentation [24], [25]. However, it appears that, for the first time, this paper presents an integrated approach to measuring spatial uncertainty in images by modulating the calculi of probability and fuzzy sets to incorporate their interdependencies. The mathematical development of the proposed method is based on the notion of the probability measure of fuzzy sets and the definition of the entropy of a fuzzy event with respect to a probability distribution that is derived from the theory of geostatistics. The novelty of this mathematical model is that the inherently fuzzy partitions of the image space can be used to model the spatial uncertainty of the image by coding the data as probability values at different degrees of membership of belonging to the fuzzy partitions. As a result, the derivations of the fuzzy sets and spatial probability measures of the image uncertainty allow the quantification of the entropy of fuzzy pixels with respect to their probability distributions. This type of a measure of uncertainty or entropy can be readily utilized as a pattern feature for image classification.

## Methods

### Entropy of a fuzzy event

Consider a Euclidean 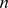-space 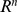 and a probability space represented by a triplet 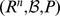, where 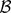 is the 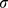-field of Borel sets (Borel sets in a topological space are the 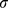-algebra generated by the open sets. An algebra of sets which is closed under countable unions is known as a 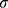-algebra, 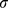-field or Borel field [26]) in 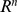 and 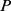 is a probability measure over 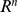. Also, let 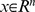 and 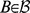. The probability of 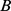, 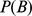, can be expressed as
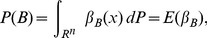
(1)where 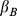, 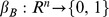, is the characteristic function of 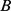, and 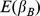 is the expectation of 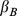.

Let 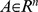 be a fuzzy set and its membership function 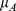, 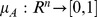, is Borel measurable. The probability of a fuzzy event 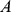 can be defined by the Lebesgue-Stieltjes integral [17] as
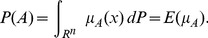
(2)


As Eq. (1) defines the probability of a crisp event as the expectation of its characteristic function, so the probability of a fuzzy event defined in Eq. (2) is the expectation of its membership function. The presented definitions of a fuzzy event and its probability constitute to the generalized framework of the theories of fuzzy sets, probability, and information [17]. Further study has also shown that the theory of probability is of a rich structure for incorporating fuzzy events within its framework to logically generate probabilities of fuzzy events so that the uncertainty of outcomes and of imprecision can be successfully unified [27].

To specifically explore how the concepts of fuzzy sets, probability and information can be made in a coherent framework; we turn the discussion to the notion of the entropy of a probability distribution, which is mathematically expressed as
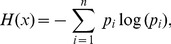
(3)where 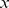 is a random variable taking values 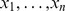 with respective probabilities 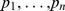, 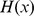 is the entropy of the distribution 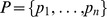, and for 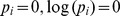, indicating the non-feasibility of obtaining information about an impossible event.

The definition expressed in Eq. (3) suggests that the entropy of fuzzy event 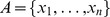 with respect to a probability distribution 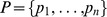 can be defined as [17]

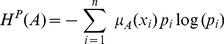
(4)where 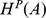 can be interpreted as the uncertainty of outcomes associated with a fuzzy event.

The above definition of the entropy of a fuzzy set forms the basis for our modeling of the spatial uncertainty of the intensity distribution in an image. It has been widely known that categorical information is inherently imprecise in images, particularly in the context of biology and medicine [14]–[31]. First, we apply the theory of fuzzy sets as a calculus for the treatment of uncertainty associated with the classification of image intensity distributions. Second, we use geostatitical tools to study the spatial continuity as a transition probability in an image to evaluate its spatial uncertainty. These two types of uncertainty measures allow fuzzy sets, probability and information to work in concert for the identification of image classes.

### Modeling image-content imprecision with the FCM algorithm

Uncertainty in an image, which is inherently due to the imprecise description of the image content, can be mathematically modeled by the partition of the image space using the fuzzy 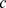-means algorithm [32]. Let an image 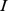 of size 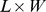 be arbitrarily partioned into a number of imprecise clusters using the fuzzy 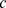-means algorithm [32]. Mathematically, let 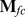 be a fuzzy 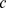-partition space, 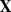 be a subset of the real 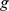-dimensional vector space 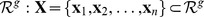 where 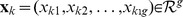. The fuzzy 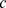-means (FCM) clustering is based on the minimization of the fuzzy objective function 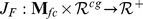, which is defined as [32]

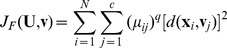
(5)where 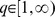 is the fuzzy weighting exponent, 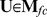, 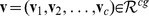, and 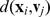 is any inner-product norm metric induced on 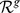.

The fuzzy objective function expressed in Eq. (5) is subject to the following constraints:
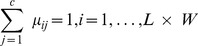
(6)where
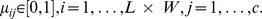



The objective function 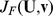 is a squared error clustering criterion and to be minimized to optimally determine 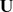, and 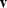. A solution to the minimization of the objective function is by a process of iteratively updating 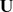 and 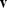 until some convergence is reached [32]. Thus, given fuzzy 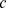 partitions, the FCM assigns each pixel to the 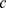 clusters with its respective membership grades. In other words, the FCM-based cluster analysis can be utilized to construct the modeling of uncertainty in an image in the context of imprecise boundaries or ill-defined classes.

### Modeling spatial uncertainty of imprecision with indicator kriging formalism

The uncertainty involved with imprecision in image intensity has been addressed with the notion of fuzziness; whereas the uncertainty regarding to pixel locations refers to spatial randomness, which can be modeled with the indicator kriging formalism of geostatistics [33]. The indicator kriging has been applied as a natural tool for determining a non-parametric conditional probability distribution of categorical data [34]. An interest in this study is to utilize indicator kriging to construct local spatial distributions of uncertainty in an image, which can be incorporated within the framework of the probability measure of fuzzy information. Let 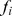 be the intensity value of a pixel located at 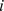, 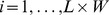, 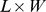 is the size of the image. Here, the purpose of applying the indicator formalism is to estimate the probability distribution of uncertainty at unsampled location 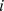. The cumulative distribution function is usually estimated with a set of cutoff thresholds 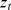, 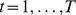; and the probabilities are then determined by coding the data as binary indicator values. The indicator coding at location 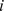 is defined as follows [34]

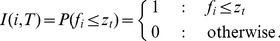
(7)


Using thresholds 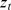, 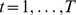, as values in the range of the image intensity does not conveniently offer a procedure for modeling spatial uncertainty in an image (for example, the thresholds can be chosen from the histogram of the data to represent percentiles, which are the values below the percentanges of the observations [35]). Therefore, instead of using 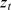, we make use of the previously discussed fuzzy image partitions or clusters 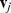, 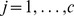, which allows every pixel to belong to every partition with different fuzzy membership grades, and apply a series of 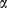-level cuts, 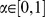, to code the categorical pixels as follows:
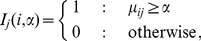
(8)where 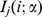 is the indicator that codes the assignment of 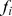 to cluster 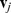 having a fuzzy membership grade being equal to or greater than 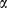. Here, 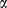 can be selected as a set of the fuzzy membership grades to represent the degrees of imprecision that indicate the possibility being higher than the most fuzzy value of 0.5.

The next step of the indicator kriging formalism is the determination of the cumulative distribution function (CDF), which characterizes the probability of 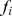 belonging to 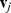 with a membership value of being greater or equal to 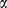, and can be mathematically expressed as
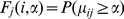
(9)


Taking advantage of the available information of 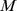-neighboring data, the conditional CDF is
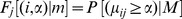
(10)where 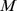 is the number of neighboring pixels of 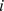.

The CDF according to the indicator expressed in Eq. (8) can be estimated using the ordinary kriging [35], and the result of indicator kriging is a model of spatial uncertainty at the pixel at location 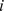:
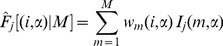
(11)where 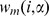 is the ordinary kriging weight that indicates the influence of neighboring pixel 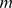 over pixel 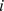 with respect to level cut 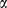. These weights can be optimally determined by the ordinary kriging system of equations [35]:
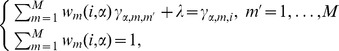
(12)where 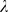 is a Lagrange multiplier, 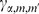, with a lag of absolute difference 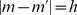, is the semivariogam of the indicator 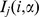 expressed in Eq. (8), and is defined as the expected value [34]:
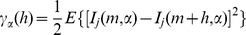
(13)


The indicator semi-variogram that is experimentally calculated for lag distance 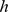 is defined as the average squared difference of values separated by 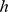:
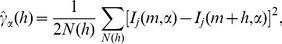
(14)where 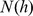 is the number of pairs for lag 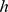.

Alternatively, the ordinary kriging system can be expressed in a matrix form as
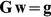
(15)where 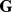 is the square and symmetrical matrix that represents the semi-variogram value between the known neighboring values 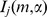, 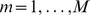; 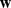 is the vector of kriging weights; and 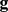 is the vector representing the semi-variogram values between 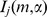 and 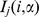, 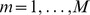, 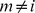. These terms are defined as [35]

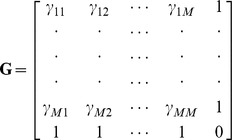
where 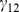 is the semi-variance of 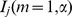 and 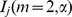;
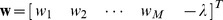
where 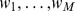 are the kriging weights, 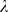 is a Lagrange multiplier, and 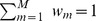; and
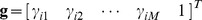
where 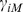 is the semi-variance of 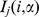 and 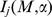.

Given that 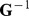 exists, the kriging weights can be obtained by solving:
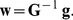
(16)


An implicit assumption of the ordinary kriging system having presented in Eq. (12) is that the underlying statistics are invariant in space under translation. Such a property is known as statistically stationary. However, statistical stationarity is a property of a random function, but not an inherent property of real data [36]. This nonstationary property is also true for medical images in which different internal organs can have different variations of the image intensity and the mean of the image changes locally. Here, kriging with a nonstationary mean is applied to enhance the reliability of the estimate of the kriging weights. This technique is called universal kriging (UK) [37], [38].

In ordinary kriging, the estimation is carried out with the error variable from a stationary mean that must be known at all positions and can be set as the global mean or modeled with a drift or a local trend. A local mean with a drift 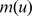 can be modeled as a linear combination of the geometric coordinates of the pixels with a local neighborhood as
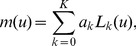
(17)where 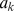 are unknown drift coefficients, 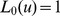 (constant function for the constant-mean case), and 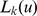, 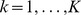, are the polynomials or basis functions, which can be modeled as the first-degree or second-degree terms as follows, respectively.
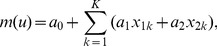
(18)

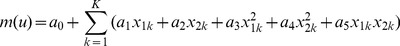
(19)where 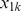 and 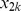 are the pixel coordinates in row-wise and column-wise of 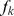, respectively.

The drift effect can be incorporated into the ordinary kriging system to find kriging weights as additional constraints. Solving this extended set of simultaneous equations, a set of universal kriging weights that model the drift within the local neighbors around the location of the unknown value. In general, the UK system can be expressed with the following matrix structure
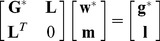
(20)where 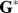, 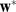, and 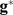 are the 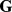 without the last row and last column, 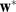 without the last row, and 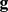 without the last row as defined for the ordinary kriging system, respectively;
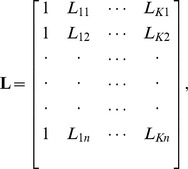
where 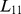 denotes 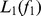,
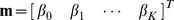
where 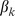, 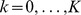 are the 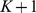 additional Lagrange multipliers, and
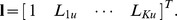



### Image classification with integrated uncertainty modeling

The integrated framework for modeling uncertainty due to both imprecision and randomness has been formulated. In other words, a new type of image feature has been introduced in terms of the probability of a fuzzy event for pattern classification. In this context, a fuzzy event can be an imprecise object or sample to be categorically identified. In application, the next task is to decide which class that best matches the feature extracted from the unknown sample. This is pattern classification that associates the appropriate class label with the test sample by using the descriptive features. A general way is to use a function or a classifier, such as a distance measure, to find the class with features that differ the least amount from the features of the unknown sample. The discussion completes with a decision or classification procedure for a computed set of entropy features for 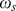, 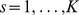, classes as follows.

Let fuzzy cluster centers 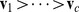, and 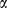-level cuts 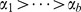 (if such orders do not exist, then the orders are rearranged). Also, let 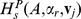, 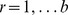, 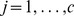, be the entropy of fuzzy event 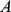 with respect to a probability distribution 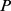 defined in Eq. (4), obtained by using 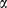-level cut 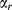 and fuzzy partition 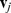 for class 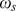:
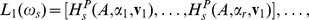


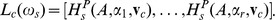



Given 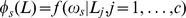, where 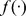 is a monotonically increasing discriminant function (the larger the value of the function the better the match); the decision for classification is carried out as follows:
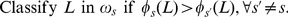
(21)


It is noted that the above decision rule expressed in Eq. (21) is general and can be applied to any type of pattern classifiers.

## Results and Discussion

### Detection of Mitochondria in Microscope Images

The proposed method was applied for the detection of mitochondria in microscope images. The mitochondrion is a membrane-bound organelle found in most eukaryotic cells. Mitochondria are considered as the powerhouse of the cell because they function as the platform for generating the production of chemical energy. The visual information of mitochondria revealed by the recent advanced technology in nanoimaging opens doors to life-science researchers to gain insights into its spatial structure and its spatial distribution within the cell. In order to simulate and model mitochondria using a large amount of images, the first task in image processing is the automated detection of this organelle. In fact, the classification of molecular images has been a long-pursued research in the disciplinary field of engineering and computer science in life sciences [39], [40]. However, there is always a strong demand for exploring appropriate feature extraction methods for the automated identification of particular types of objects or regions of interest in cell biology with different levels of technical challenge, ranging from the detection of cells nuclei [41] to subcellular patterns [2]. If different types of the images can be automatically distinguished by computerized methods, such an ability can help researchers to quickly and accurately study cell function to discover mechanisms underlying complex diseases, and carry out spatial modeling and simulation of biological signaling pathways, which may identify critical organelles attributing to the regulation of the cellular process within the intracellular space.

**Figure 6:**
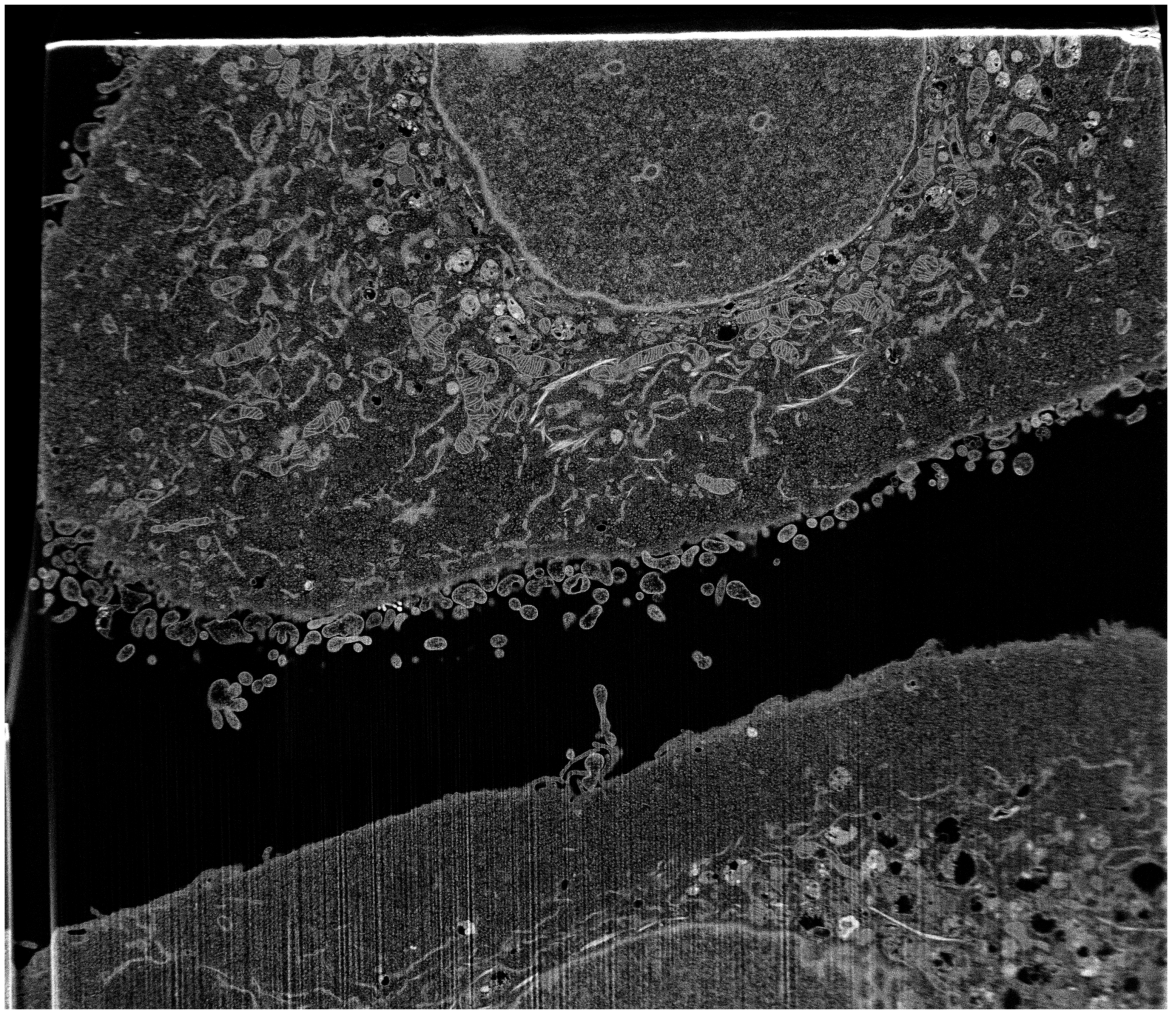
An FIB-SEM image of SCC-61 (cancer cell).

**Figure 7:**
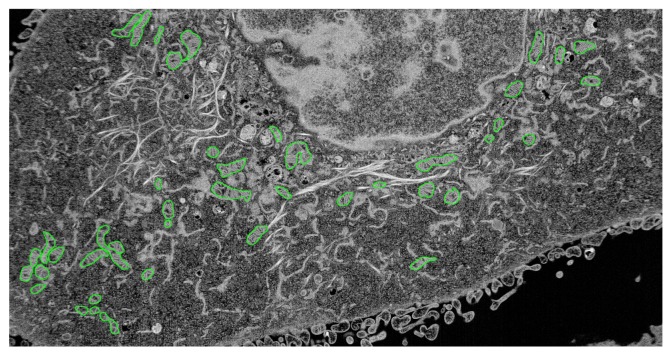
An FIB-SEM image of SCC-61 (cancer cell), in which the edges of ground-truth mitochondria were marked by a cell biologist.

The cells were imaged using scanning electron microscopy (SEM) and focused ion beam (FIB) technology with Helios NanoLab 650, which is one of the most recent advances in field emission SEM and FIB technologies and their combined use, and designed to access an extremely high resolution characterization, and higher quality sample preparation. Figure 6 is a typical SEM-FIB image showing half of the intracellular space of a cancer cell line that was derived from a human head and neck squamous cell carcinoma (SCC-61) parental line [42]. Figure 7 shows a typical FIB-SEM image of the same cancer cell in which the ground-truth mitochondria were manually identified and marked by a cell biologist. The interest here is to detect image regions of interest that contain the mitochondria. Such detected regions will greatly alleviate the difficulty in the image segmentation of the mitochondria [43] to facilitate the spatial modeling and simulation of the role of this major organelle for studying human complex diseases such as cancer [44], [45].

The detection of the mitochondria in the intracellular space was carried out with a window of 53 by 60 pixels, which is the average size of the mitochondria in the images. The number of clusters 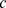 and the exponent 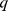 expressed in Eq. (5) were selected to be three (to approximately represent the number of intensity groups in the images) and two (commonly specified in many applications), respectively. The values of the 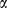-cut used for the indicator expressed in Eq. (8) are 0.5, 0.6, 0.7, 0.8 and 0.9. The numbers of the neighboring pixels 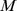 used in Eq. (11) are 5 and 7. The detection of the mitochondria was performed by moving the 53-by-60 window along the horizontal and vertical directions of the image to extract different features for training. Twenty scans of the FIB-SEM images of the cancer single cell were available in this study. To show the effectiveness of various feature extraction methods, only one image was used for training. The training was performed by extracting the proposed probabilistic entropy measure of the fuzzy information, expressed in Eq. (4), of the mitochondrial and non-mitochondrial regions using OK, denoted as PEFI1, and expressed in Eq. (12), and using UK, denoted as PEFI2, expressed in Eq. (20), respectively. To compare with other feature extraction methods, the same images were used to obtain the gray-level co-occurrence matrix (GLCM), fractal dimension (FD), semi-variogram (SV), semi-variogram exponent (SVE), and the indicator-kriging co-occurrence matrix (IKCM) for the mitochondrial and non-mitochondrial objects, described in [46]. Ten lags were used to extract the semi-variogram values of each image window. If the image window contained whole or part of a mitochondrion, it was labelled as a mitochondrial object. This is designed to capture all small regions of interest containing the mitochondria in order to maximize the sensitivity (true positive rate), while the specificity (true negative rate) can be first reasonably obtained and then maximized in the localized image segmentation task performed window by window. To validate the effectiveness of the extracted features, two simple measures that are the Euclidean and Mahalanobis distances were used to calculate the similarity between the unknown (test) samples and the trained prototypes of the mitochondrial and non-mitochondrial objects.

Sensitivity and specificity are statistical measures of the performance of a binary classification test. In this study, the sensitivity (true positive rate) is the percentage of the mitochondrial regions that are correctly identified; whereas specificity (true negative rate) is the percentage of the non-mitochondrial regions that are correctly identified. Tables 1 and 2 show the sensitivity and specificity of the experiment obtained from several feature extraction methods using the Euclidean and Mahalanobis distances, respectively. In general, the SV, SVE, IKCM, PEFI1 and PEFI2 performed consistently using either the Euclidean distance or Mahalanobis distance. The PEFI2 yields the best results in both sensitivity and specificity in both distance measures: sensitivity  = 100% and specificity  = 93%, using the Euclidean distance; sensitivity  = 100% and specificity  = 97%, using the Mahalanobis distance. The PEFI1 yields the second best: sensitivity  = 100% and specificity  = 91%, using the Euclidean distance; sensitivity  = 100% and specificity  = 94%, using the Mahalanobis distance. Using the Euclidean distance, the GLCM achieved 100% for sensitivity, but its specificity is lowest (5%) in comparisons with the other features. On the other hand, the FD performed well with the specificity (90%) but poorly with the sensitivity (35%), using the Euclidean distance. In general, the use of the Mahalanobis distances improved all the detection results provided by all the features, in which the specificity obtained by the GLCM (65%) is significantly higher than using the Euclidean distance.

**Table 1:** Sensitivity and Specificity of Mitochondrial Detection Obtained from Various Features using Euclidean Distance.

	GLCM	FD	SV	SVE	IKCM	PEFI1	PEFI2
Sensitivity	100	35	95	100	100	100	100
Specificity	5	90	70	50	87	91	93

**Table 2:** Sensitivity and Specificity of Mitochondrial Detection Obtained from Various Features using Mahalanobis distance.

	GLCM	FD	SV	SVE	IKCM	PEFI1	PEFI2
Sensitivity	100	80	100	100	100	100	100
Specificity	65	90	70	60	94	94	97

### Identification of Abdominal Tissues on Computed Tomography

The proposed method was also tested for abdominal wall hernia mesh tissue classification on computed tomography (CT), which was recently carried out in [47]. The data were obtained from the abdominal and pelvic CT scans of patients. Eight types of mesh were studied in this experiment: alloderm (M1), marlex (M2), parietex (M3), proceed (M4), strattice (M5), surgimend (M6), and surgisis (M7), and permacol (M8). The available numbers of M1 = 10, M2 = 9, M3 = 30, M4 = 34, M5 = 18, M6 = 15, and M7 = 7, M8 = 54. Half of the samples of each mesh type were used for training and the other half for testing. The classification was carried out by the 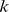-nearest neighbor method [48], where 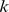 = 3 to decide which type of mesh was present. Ten Monte-Carlo iterations were used for the random selection of training and testing data to enhance the statistics of the experimental results.

The results obtained from the feature extracted by the proposed spatial uncertainty modeling (SUM) were compared with other features extracted by wavelets, the gray-level co-occurrence matrix entropy (GLCME), geostatistical entropy (GE), probabilistic Fusion (PF), and entropy fusion (EF) models, which were carried out in [47]. Further details about the CT data and implementations of GLCME, GE, and EF methods were described in [47]. For the implementation of the FCM, the number of clusters 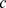 and the exponent 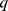 expressed in Eq. (5) were selected to be three (to approximately represent the organs of gray and white intensities and the background) and two (commonly specified in many applications), respectively. The values of the 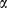-cut used for the indicator expressed in Eq. (8) are 0.5, 0.6, 0.7, 0.8 and 0.9. The numbers of the neighboring pixels 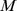 used in Eq. (11) are 5 and 7. The total average results obtained from the current technique, using OK (PEFI1) and UK (PEFI2), and other methods are shown in Table 3. The results show that the performance of proposed feature extracted by PEFI1 (94.50%) and PEFI2 (94.92%) are the best among the other features for the classification task. The performance of PEFI2 is only slighly better than that of PEFI1. It should be noted that the proposed feature not only outperforms the other individual features (wavelets, GLCME, and GE), but also yields better classification rates than the combinations of the GLCM and geostatistical models in terms of entropy (92.81%) and probability (90.76%) measures.

**Table 3:** Total Average Classification Rates Provided by Different Methods.

Classifier	Accuracy (%)
Wavelets	45.29
Gray-Level Co-Occurrence Entropy (GLCME)	56.61
Geostatistical Entropy (GE)	76.72
Probabilistic Fusion (PF)	90.76
Entropy Fusion (EF)	92.81
PEFI1	94.50
PEFI2	94.92

### Classification of Logos on Document Imaging

Furthermore, the proposed spatial uncertainty modeling approach was tested for the classification of logos on document images. Ten sets of logos were obtained from the public-domain logo database of the University of Maryland, which consist of 105 intensity logo images. Fifty other logo images were also included, embedded in several document formats including letters, faxed documents, and billing statements [49], [50]. All logo images were also generated subject to translation, scaling, orientation and degradation to create different sets of images [49], [50]. Image rotations include 2-degree, 4-degree, 6-degree, 8-degree, 10-degree orientations. The images were shrunk by the factor of two using the bicubic interpolation and anti-aliasing. For the translation, all images were shifted left (x-shifted) by 50 pixels and up (y-shifted) by 30 pixels. All images were degraded with Gaussian noise of zero mean and 0.02 variance.

The features extracted from the logo images are: 1) semi-variograms, 2) Zernike moments, 3) wavelets, 4) Gabor features, and 5) SUM-based feature. The first four features were studied in [50]–[52]. These features were equally divided into training and test datasets. For the implementation of the FCM expressed in Eq. (5), the number of clusters 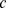 = 2 to represent the object and background, and the fuzzy exponent 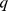 = 2. The values of the 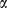-cut used for the indicator expressed in Eq. (8) are 0.5, 0.6, 0.7, 0.8 and 0.9. The numbers of the neighboring pixels 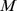 used in Eq. (11) are 5 and 7.

The datasets were equally split into half for training and the other half for testing. Furthermore, the data were randomly selected for 10 times to repeat the training and testing in order to establish statistically meaningful results of the experiment. The 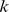 nearest neighbor (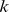-NN) method was applied for the task of classification, with 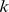 = 3, 5, and 7. The total average classification results shown in Table 4 suggest that the proposed SUM-based feature outperforms the use of the other four features, with the order of performance from the lowest to highest classification rates as follows: wavelet feature, Gabor features, Zernike moments, semi-variograms, and proposed feature (PEFI1 and PEFI2, where both algorithms achieved an equal classification rate).

**Table 4:** Total Average Logo-Image Classification Rates (%).

Semi-variogram	Zernike	Wavelets	Gabor	PEFI1	PEFI2
69.77	61.50	47.40	50.21	72.89	72.89

## Conclusion

A modeling of spatial uncertainty in images for pattern classification using the theories of fuzzy sets and geostatistics has been presented and discussed. The proposed model has been implemented as a new feature extraction method for the classification of image patterns.

The entropy of a fuzzy image information with respect to a probability distribution is calculated as an integrated spatial uncertainty of the image, which can be used for characterizing categorical images. Simple classifiers were trained with this new feature for comparions with other related existing features. The training of the proposed feature with advanced classifiers can be expected to enhance the results. In particular, the applications of the proposed approach for automated detection of mitochondria in the real intracellular imaging of a cancer cell line, tissue identification and logo classification have been carried out. The comparative results suggest the usefulness of the proposed mathematical framework for image feature extraction. Being similar to the use of the probabilities of the gray-level co-occurrence matrix, the indicator-kriging probabilities can be utilized to construct other statistical features of an image for pattern classification.

The model developed in this study can be further improved by selecting effective strategies for selecting the number of fuzzy clusters and adding additional spatial constraints to the fuzzy objective function. In particular, as the constrained independent component analysis has been developed to reduce ambiguity in studying fMRI data by imposing temporal and spatial constraints to the mathematical model [53]; the fuzzy objective function defined in Eq. (5) can be modified by adding similar temporal and spatial constraints [54], [55] to improve the modeling of uncertainty in the setting of geostatistics.
